# High Throughput Screening for Anti–*Trypanosoma cruzi* Drug Discovery

**DOI:** 10.1371/journal.pntd.0003259

**Published:** 2014-12-04

**Authors:** Julio Alonso-Padilla, Ana Rodríguez

**Affiliations:** New York University School of Medicine, Department of Microbiology, Division of Parasitology, New York, New York, United States of America; Universidad Autónoma de Yucatán, Mexico

## Abstract

The discovery of new therapeutic options against *Trypanosoma cruzi*, the causative agent of Chagas disease, stands as a fundamental need. Currently, there are only two drugs available to treat this neglected disease, which represents a major public health problem in Latin America. Both available therapies, benznidazole and nifurtimox, have significant toxic side effects and their efficacy against the life-threatening symptomatic chronic stage of the disease is variable. Thus, there is an urgent need for new, improved anti–*T. cruzi* drugs. With the objective to reliably accelerate the drug discovery process against Chagas disease, several advances have been made in the last few years. Availability of engineered reporter gene expressing parasites triggered the development of phenotypic in vitro assays suitable for high throughput screening (HTS) as well as the establishment of new in vivo protocols that allow faster experimental outcomes. Recently, automated high content microscopy approaches have also been used to identify new parasitic inhibitors. These in vitro and in vivo early drug discovery approaches, which hopefully will contribute to bring better anti–*T. cruzi* drug entities in the near future, are reviewed here.

## Introduction

Chagas disease, caused by the protozoan parasite *Trypanosoma cruzi*, is endemic to Central and South America [1], [2]. Its impact represents the largest burden due to a parasitic disease in the Western hemisphere [2]. Estimates point at 20,000 deaths per year; over 10 million people currently infected, many of them unknowingly; and another 100 million living in risk-of-transmission areas [3]. The infection is transmitted by hematophagous triatomine insect vectors (Reduviid order) that easily proliferate in settings with poor housing conditions, rendering those with lesser economic resources most vulnerable to the infection [2], [4]. The parasite cycles between insect vectors and mammalian hosts in four distinguished parasitic life-stages: replicative epimastigotes and host infective metacyclic trypomastigotes in the insect, whereas free-swimming trypomastigotes and intracellular replicative amastigotes are found in the host [4].

The disease starts with a short acute phase, during which blood parasite levels are relatively high and sufficient for successful conventional diagnosis. Although mostly asymptomatic, this first stage can cause death in approximately 5% of acutely infected patients [4]. An indeterminate “silent” stage that may span more than 20 years follows, during which, despite pathogen persistence, parasitemia is hardly detectable and no major clinical symptoms are encountered [4]. Many of the indeterminately infected people clear the infection or just never present clinical symptoms; however, about 30% of the chronically infected patients suffer, at some point, severe heart and gut tissue damage that, if untreated, ultimately leads to total disability and death [2], [4].

With no vaccine in view, the treatment of Chagas disease relies on chemotherapy with benznidazole (BNZ; LAFEPE, Pernambuco) and nifurtimox (NFX; Lampit, Bayer Healthcare, Leverkusen), which have been in use for the last 40 years and have failed to control the disease [5]. Long-term dosage regimes are required for effective cure; however, severe side effects often prompt the discontinuation of the treatment [5]. Both drugs show good efficacy against the short and mostly asymptomatic acute stage and are also indicated for treatment during the early chronic phase and to prevent congenital transmission [6]. In the chronic phase, the efficacy of these drugs can vary because of reasons like patient age, the time the patient has had the disease, and the criteria used to define parasite clearance. Although there is not yet agreement about the efficacy of these drugs in the chronic phase [2], recent reports demonstrate significant efficacy and propose that treatment should be administered at all disease stages [7]–[11]. Changes in the long dosage regimes and/or combinatorial therapies have also been suggested to avoid the severe side effects of the current available drugs [12]. In any case, new therapeutic options are much awaited, not only due to toxicity but also because susceptibility to BNZ and NFX among different *T. cruzi* strains/isolates is variable [5]. However, the current clinical trials portfolio against Chagas disease is populated with modifications on the use of the already available BNZ and NFX (Table 1) and two re-purposed antifungal azoles. The latter compounds held great promise as potent inhibitors of the parasite fundamental ergosterol biosynthetic pathway [21]. Unfortunately, despite sporadic successful outcomes [22], the Phase II clinical trials performance of posaconazole and the ravuconazole derivative E1224 have been disappointing [23]–[25].

**Table 1:** Current status of clinical trials against Chagas disease.

Drug	Current use	Trialed for	Target	Stage (notified status)	Ref.
BNZ	Early and acute Chagas	Chronic Chagas cardiomyopathy	DNA damage	Phase III BENEFIT trial of a cohort of 1,000 patients that includes healthy volunteers (unknown).	[13]
NFX	Early and acute Chagas	Bioequivalence of two drug formulations	DNA damage	Phase I (recruiting).	[14]
POS	Antifungal	All stages	CYP51	Phase II (completed).	[15]
POS	Antifungal	Asymptomatic chronic stage	CYP51	Phase II Merck Sharp & Dohme Corp. sponsored trial, includes a combined POS + BNZ test group (ongoing but not recruiting).	[16]
E1224	Ravuconazole antifungal pro-drug awaiting approval	All stages	CYP51	Phase II DNDi sponsored study in Bolivia (unknown).	[17]
Amiodarone	Heart arrythmia	Symtomatic Chagas cardiomyopathy	Inhibition of ergosterol synthesis and Ca^2+^ homeostasis disruption	Use of amiodarone as comparator of implantable cardioverter defibrillator (ICD). Phase not specified (not yet recruiting).	[18]
Carvedilol	Heart failure	Symtomatic Chagas cardiomyopathy	Beta-blocker	Phase IV to evaluate safety and efficay after renin angiotensin system inhibitors administration (completed).	[19]
Bisoprolol	Alleviate progression to heart failure	Symtomatic Chagas cardiomyopathy	Beta-blocker	Phase III (completed).	[20]

Search efforts for new anti–*T. cruzi* drugs stand upon the advancements achieved in the past years with the use of engineered reporter parasites for in vitro phenotypic assays [26]–[28]; reliable, quick in vivo protocols [28], [29]; as well as the application of cutting-edge digital imaging technology like high-content microscopy [30]–[32]. In the search of new chemotherapeutics against neglected diseases, in which very few validated targets exist, non-reductionist, whole cell phenotypic approaches hold significant advantages [33]. Phenotypic screening represents a cost-effective method to identify previously unknown targets and provide a wider view of the antiparasitic drug activity that can be hitting either single or multiple targets [33], or even be associated to host factors [34]. Another advantage is that whole cell screening against obligate intracellular parasites yields a straightforward functional perspective of the cell membrane permeability of the compounds [33]. Reliable, reproducible high throughput screening (HTS) phenotypic assays are of great benefit to *T. cruzi* drug discovery, where assay costs become an issue to be taken into consideration because of the low discontinuous funding schemes dedicated to neglected diseases research and the lack of interest in these diseases by large pharmaceutical companies. For the same reason, the development of cost-effective in vivo tests to further progress the HTS-retrieved hits is of great importance [29], [35], [36]. The availability of transgenic parasites, combined with the development of small animals imaging platforms, has already positively impacted the early drug discovery against *T. cruzi*, and will be of great importance to feed the drug discovery pipeline for Chagas disease in the near future [35].

## Methods

Literature searches for this work were made in PubMed under the terms “anti–*Trypanosoma cruzi* assays,” “image-based parasites,” and further following the citations in related references. Information on clinical trials was obtained from the website http://clinicaltrials.gov and updated press releases were queried on general web browsers. Performance and outcome of the NIH chemical collection high throughput screening against *T. cruzi* made at the Broad Institute was checked at the online-available book *Probe Reports from the NIH Molecular Libraries Program*.

## In Vitro Phenotypic Assays to Identify New Anti–*T. cruzi* Drugs

### HTS assays based on recombinant reporter parasites

An increasing knowledge of *T. cruzi* molecular biology and the development of plasmids and efficient parasite transfection protocols [37] permitted the construction of reliable and robust transgenic *T. cruzi* parasites of multiple genetic backgrounds [26], [28], [37], [38]. The availability of these microorganisms transformed the previously time-consuming, labor-intensive, observer-biased visual microscopic counting, into faster, unbiased colorimetric, fluorometric, or luminescence-based measurements, which have made feasible the screening of large compound libraries. Transgenic *T. cruzi* parasites that express bacterial β-galactosidase reporter enzyme [26], firefly luciferase [28], [38], or the tandem tomato fluorescent protein [28] have been made available to the scientific community. As part of their development as tools for anti–*T. cruzi* inhibitory assays, these genetically engineered microorganisms were shown to biologically perform like their wild-type counterparts, in terms of growth, cycle stage transitions, mice infection, and in vitro sensitivity to known drugs [26], [28]. For the recombinant parasites carrying either the β-galactosidase reporter gene or the firefly luciferase gene, a substrate addition step is required and a single end-point measurement of the reporter enzyme activity can be obtained. To detect β-galactosidase activity, colorimetric and luminescent substrates have been used (respectively, chlorophenol-red-β-D-galactopyranoside, Sigma-Aldrich [26], and GalScreen, Life Technologies [39]). It must be noted that colored compounds, which are frequently found in chemical libraries, might interfere with the readout when using colorimetric reporters [26]. This issue can be addressed by using luminescent or fluorescent-based readouts as in the first HTS campaign performed against *T. cruzi* at the Broad Institute [39] and in the more recent HTS made at GSK [40]. Another reason for preferring luminescence and/or fluorescence detection methodologies is their higher sensitivity, which allows for greater miniaturization of assays. An important issue to be considered in the use of transgenic parasites in the screening of chemical libraries is that reporter activity inhibitors (i.e., chemical compounds that inhibit luciferase or β-galactosidase) are selected as hits. Thus, secondary assays, such as image-based assays that rely on non-recombinant strains, are needed to identify these false hits.

Firefly luciferase–expressing parasites require the addition of luciferin before performing the readout [28], [38]. In contrast, the important advantage offered by the self-fluorescent recombinant parasite strains developed in Prof. Tarleton's lab resides on their substrate-independent detection, which allows a continuous activity measurement [28]. This property appears particularly important to investigate parasite growth kinetics, which could provide valuable insights of compounds' mechanisms of action and, for example, aid to prioritize those with high parasite lytic activity. However, the higher sensitivity of detection in vivo of methods such as PCR of specific tissues compared to luminescent or fluorescent parasites would make these methods more useful in the detection of small populations of parasites, for example, in chronically infected animals.

Recombinant *T. cruzi* parasites have already been used in a series of in vitro assays designed to detect drug susceptibility mainly of the intracellular amastigote life-stage [26]–[28], the parasite replicative form in the mammalian host and the preferred parasitic target stage. When screening a molecular library against intracellular amastigotes, parallel host cell toxicity assays on the retrieved hits or “active” compounds are necessary to determine whether the activity is specifically antiparasitic or either totally or partially due to a disruption of host-cell biology [27], [41], [42]. The selectivity index is then calculated using the relative IC_50_ values of the host cell and the parasite.

**Figure 1:**
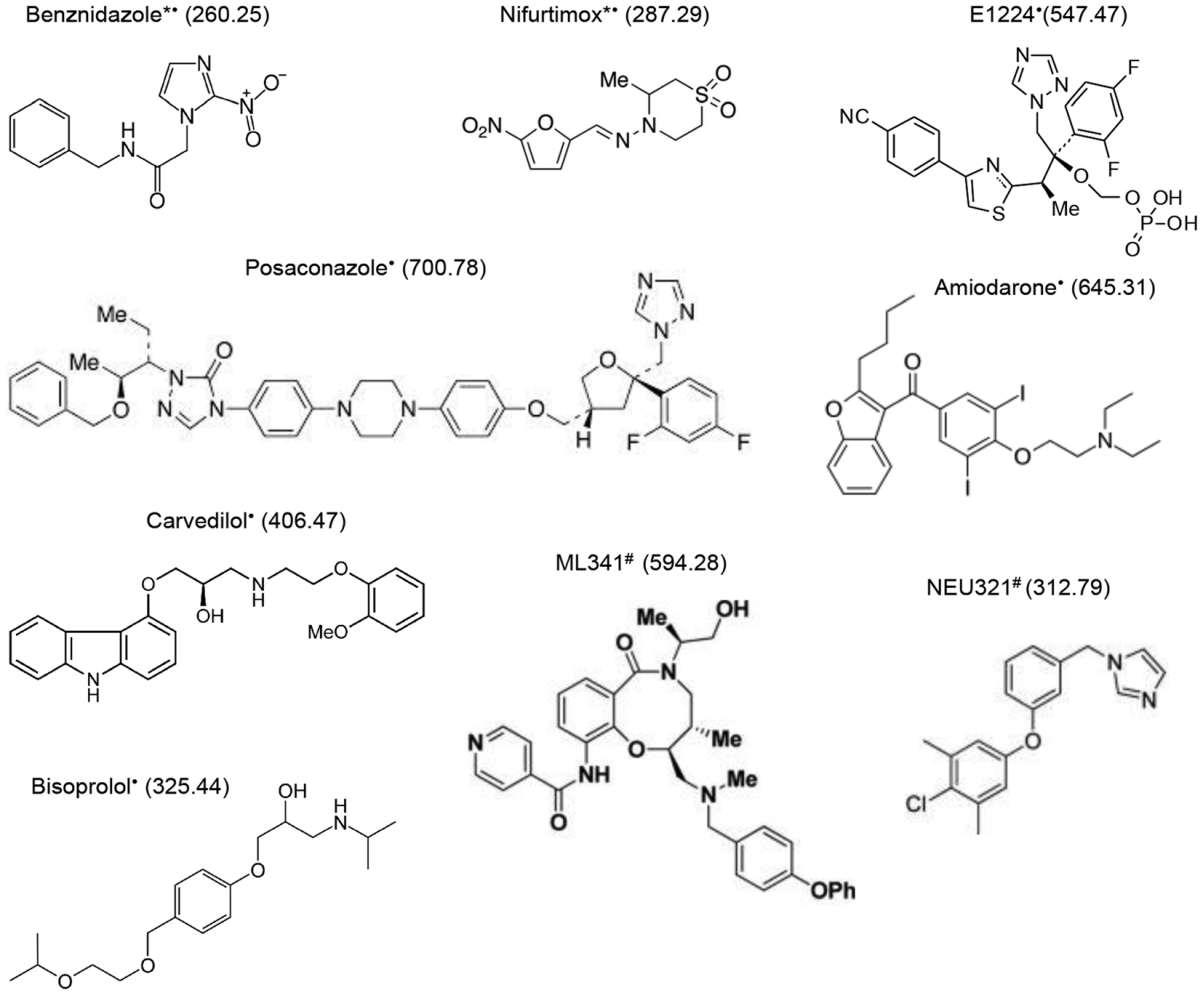
Anti–*Trypanosoma cruzi* compounds. Chemical structures of anti–*T. cruzi* compounds currently in clinical use (*) and currently or recently in clinical trials (•), and of the most promising compounds retrieved out of the Broad Institute HTS campaign [44] and the screening of a smaller, diversity-oriented chemical library [39], [43] (#).

The most important impact of the developed in vitro assays based on transgenic parasites has been their amenability to HTS [39], [40]. In terms of impact, the adaptation of the *T. cruzi* β-galactosidase–based assay [26] to HTS standards established a milestone in the anti-Chagas early drug discovery process. After a protocol improvement to reduce the assay time, but still at its original 96-well plate format, the assay showed its reliability in the screening of a 2,000 compounds chemical library [27]. The β-galactosidase reporter assay was developed into a 384-well format with a luminescent readout allowing higher throughput for the HTS campaign at the Broad Institute, where screening of 303,224 compounds (the NIH library) yielded 4,394 hits. After the confirmation of activity and determination of efficacy and cytotoxicity of the hits, 3,005 compounds with an IC50 <10 µM and>10-fold selectivity against the parasite versus the host cells were selected [39]. All information, including the chemical structures of the hits, is publicly available at Pubchem (AID 1885). Further analysis of the hits selected by this HTS revealed that a high percentage of them were not reproducible in a similar assay. It is likely that the reason for such discrepancies resided in the quality of the chemical compounds used for the original HTS [41]. Another HTS of a diversity-oriented synthesis chemical library of 77,312 compounds has been performed [42]. The most attractive confirmed hits in terms of potency, selectivity, and predicted drug-like physicochemical properties between these two HTSs were picked up for further development and the data obtained from them have served as the basis for different initiatives (Figure 1) [43]–[46]. The access to high-quality large chemical libraries will be of fundamental importance for the advance of drug discovery against neglected diseases, as it will be to get the resources and know-how to mine and test them. In this perspective, collaboration between pharmaceutical companies and academic laboratories becomes a key aspect for the successful development of future drugs.

### Image-based HTS assays

High content screening (HCS) allows the automated acquisition and analysis of multiple cellular features simultaneously [30], [46]. HCS is a powerful screening tool, the use of which has expanded in the last years because of high assay throughput, automatic image acquisition and analysis provided by user-friendly software interfaces and the possibility of robotization [30], [46]. The availability of this technology has already permitted the development of image-based assays to identify active compounds against *Leishmania donovani* [34], [47]–[49]. Similarly to HTS based on recombinant reporter parasites, automatic quantification is not biased by the observer visual parasite counting, as it is performed by an image analysis script specifically developed for this purpose [31], [32], which once set up has been shown to be extremely useful, providing very accurate readings and saving valuable time [50]. In the drug development process against *T. cruzi*, this methodology holds a clear advantage compared to reporter-based assays as it offers more versatility, which may allow the use, upon assay adaptation, of any parasite strain that is adapted for infection in vitro (including difficult-to-engineer strains and relevant clinical specimens). Additionally, this assay provides the information on host cell cytotoxicity of each compound in a single experiment, delivering immediate results on the selectivity index. An image-based assay developed by Engel and coworkers that has already been used to screen a FDA-approved drugs library (>900 compounds) where 55 hits were identified [31], and at least two other image-based assays have been referenced in perspective articles [33], [36]. More recently, scientists from Institute Pasteur in Korea have developed an algorithm to aid in the image analysis process of a *T. cruzi* infection drug inhibitory assay [32].

There are yet some disadvantages related to HCS that need to be improved, like the storage and handling of the high amount of data generated and the speed of both picture acquisition and computing analysis that can have an impact on the developed assays HTS scale up [46], [51]. A drawback specific to the reported anti–*T. cruzi* image-based assay is that it does not allow serial readouts, since cells must be fixed and stained at a single end-point. This is similar to β-galactosidase and luciferase reporter-based assays; however, in the imaging assays, the possibility to measure different cell biology features (high-content microscopes offer up to four laser channels and there is a large spectrum of available fluorophores and biological probes) in a single experiment should grant its further development. Besides the identification of new hits, HCS could also provide a major input in the hit-to-lead-optimization process and play a key part in deciphering new mechanisms of action [30], [51]. In the future, fluorescently tagged ligands or specific staining with antibodies could be incorporated in the assays to provide mechanistic information of compounds.

### In vivo intermediate throughput screening by image acquisition and shortened protocols

A fundamental step in the drug discovery path is the selection and qualification of hits to be progressed into leads [52]. A requirement for this upgrade is consistent efficacy in animal models [29], [52]. Although various animal infection models have been studied in Chagas disease research, by far the most used has been the mouse model [53]. The mouse is the most preferred in vivo model because it adequately reproduces the main pathogenic features of human *T. cruzi* infection, there is accumulated knowledge about its genetics and physiology, a large amount of reagents and tools developed to work with it, and the ease of handling and storage of colonies.

Transgenic parasites have also been used with success for in vivo studies. Bio-imaging of mouse models infected with transgenic *T. cruzi* strains (either luciferase or fluorescent recombinant parasites) allows a rapid assessment of the infectious disease process within the animals [28], [38], [41]. Before the implementation of transgenic parasites and in vivo imaging techniques, animals had to be killed to obtain end-point data on parasite detection and organ dissemination, which was very labor-intensive, presented risk for human infection, and required the use of large animal groups, all contributing to increased costs [53], [54]. Furthermore, the techniques used (PCR, in situ hybridization, and microscopic blood or tissue sections parasitic counting) were cumbersome and presented certain limitations [38]. By means of luminescent or fluorescent transgenic parasites and the appropriate detection equipment, it is now possible to accurately evaluate in vivo the infection process and the drug responses that might inhibit it. In comparison with measurements relying on sacrifice, bio-imaging has the great advantage of providing continuous readouts on anesthetized animals, which has greatly contributed to the understanding of infection dynamics and parasite tropism [55], [56]. Interestingly, *T. cruzi* chronic infections in mice were focal but moved within the body from week to week. Parasites did not localize to the heart, despite these mice developing miocarditis and cardiac fibrosis. *T. cruzi* was found in other organs and consistently in the gastrointestinal tract [56].

Another advantage of these experimental infections for drug development is that, since progression of infection can be followed in live animals, the total number of mice required for the experiments can be drastically reduced, therefore following the 3R principle (Replacement, Reduction, and Refinement) [57], and contributing to reducing research costs [38], [54]. With such advantages, in vivo protocols have already been developed [28], [29], [38], [41], [54] and are being used to test in vitro–selected hits with the best drug-like characteristics [28], [41]. Early tagging of hits in the drug development path as *in vivo–*active, i.e., prior to medicinal chemistry modifications, represents considerable savings in time and funding, which are much needed to further develop the selected compounds into drugs for a disease with serious funding shortages [29]. Some of these assays are available to the community through a fee-for-service, non-profit core facility [58].

Bio-imaging assays normally model the initial acute-phase infection of Chagas disease and would select for fast-acting compounds [54]. Since the parasite detection threshold for *T. cruzi* in bio-imaging assays normally falls around 1,000 parasites [28], [41], [56], to determine whether a particular treatment, administered either in acute or chronic phase, results in sterile clearance, chemically induced immune suppression [59] should be used after treatment to confirm it [54].

The throughput of some in vivo assays [28] can be considered intermediate, since rapid two-day assays in low number of animals (fewer than 3 per group) can be used for screening hundreds of compounds in short periods of time [29].

## Concluding Remarks

The last few years have seen a revolutionary improvement of the in vitro and in vivo screening methodologies to search for new anti-Chagas therapies based on the development of transgenic *T. cruzi* parasites engineered to constitutively express reporter genes and on the access to imaging technology that can largely ease up the process. These advances have granted the availability of robust in vitro assays suitable for high-throughput screening, as well as providing the necessary tools to design fast and reliable in vivo testing protocols. Their synergy is aimed at accelerating the early drug discovery path against a disease that has waited a long time for better therapeutics.

Very scarce economical resources are dedicated to find new drugs against neglected diseases, and therefore discovery campaigns must necessarily be cost-effective. However, the prospects are promising because the necessary tools are ready to be used and big pharmaceutical and public–private partnerships have shown their interest in the screening of large compound libraries [60], some of which are already under development [40]. It is expected that a substantial number of hits will sprout from these screens in the near future. Solid funding commitments at national and international levels need to be sustained to achieve the goal of a nontoxic, short-course, effective cure for chronic Chagas disease.

**Key Learning Points**

- Genetically engineered parasites have had a revolutionary impact in the development of both high-throughput amenable assays to screen large chemical collections for drug discovery in vitro and in vivo screening protocols to rapidly progress hit compounds.
- High technology equipment, such as automated plate readers, high-content microscopy, and luminescence imagers for live animals, has been applied to accelerate new anti-parasitic drugs search, providing high-quality readouts and reducing drug development costs.
- Collaborative frameworks between academia and the private pharmaceutical industry are necessary to bring together parasite biology and drug discovery expertise to allow the translation of academic research into medicines.

**Top Five Papers**

1. Buckner FS, Verlinde CL, La Flamme AC, Van Voorhis WC (1996) Efficient technique for screening drugs for activity against Trypanosoma cruzi using parasites expressing beta-galactosidase. Antimicrob Agents Chemother 40: 2592–2597.
2. Bettiol E, Samanovic M, Murkin AS, Raper J, Buckner F, et al. (2009) Identification of three classes of heteroaromatic compounds with activity against intracellular Trypanosoma cruzi by chemical library screening. PLoS Negl Trop Dis 3: e384.
3. Broad Institute (2009) Luminescence cell-based/microorganism primary HTS to identify inhibitors of Trypanosoma cruzi replication. PubChem BioAssay AID 1885. Available: http://pubchem.ncbi.nlm.nih.gov/assay/assay.cgi?aid=1885. Accessed 5 November 2014.
4. Canavaci AM, Bustamante JM, Padilla AM, Perez Brandan CM, Simpson LJ, et al. (2010) In vitro and in vivo high-throughput assays for the testing of anti-Trypanosoma cruzi compounds. PLoS Negl Trop Dis 4: e740.
5. Engel JC, Ang KK, Chen S, Arkin MR, McKerrow JH, et al. (2010) Image-based high-throughput drug screening targeting the intracellular stage of Trypanosoma cruzi, the agent of Chagas disease. Antimicrob Agents Chemother 54: 3326–3334.
